# Small Intestinal Intraepithelial TCRγδ^+^ T Lymphocytes Are Present in the Premature Intestine but Selectively Reduced in Surgical Necrotizing Enterocolitis

**DOI:** 10.1371/journal.pone.0099042

**Published:** 2014-06-06

**Authors:** Jörn-Hendrik Weitkamp, Michael J. Rosen, Zhiguo Zhao, Tatsuki Koyama, Duke Geem, Timothy L. Denning, Michael T. Rock, Daniel J. Moore, Melissa D. Halpern, Pranathi Matta, Patricia W. Denning

**Affiliations:** 1 Department of Pediatrics, Vanderbilt University School of Medicine and Monroe Carell Jr. Children's Hospital at Vanderbilt, Nashville, Tennessee, United States of America; 2 Division of Pediatric Gastroenterology, Hepatology, and Nutrition, Cincinnati Children's Hospital Medical Center, Cincinnati, Ohio, United States of America; 3 Department of Biostatistics, Vanderbilt University School of Medicine, Nashville, Tennessee, United States of America; 4 Center for Inflammation, Immunity, and Infection, Institute for Biomedical Sciences, Georgia State University, Atlanta, Georgia, United States of America; 5 Department of Pediatrics and Steele Children's Research Center, University of Arizona, Tucson, Arizona, United States of America; 6 Department of Pediatrics, Emory University School of Medicine, Atlanta, Georgia, United States of America; University of Florida, United States of America

## Abstract

**Background:**

Gastrointestinal barrier immaturity predisposes preterm infants to necrotizing enterocolitis (NEC). Intraepithelial lymphocytes (IEL) bearing the unconventional T cell receptor (TCR) γδ (γδ IEL) maintain intestinal integrity and prevent bacterial translocation in part through production of interleukin (IL) 17.

**Objective:**

We sought to study the development of γδ IEL in the ileum of human infants and examine their role in NEC pathogenesis. We defined the ontogeny of γδ IEL proportions in murine and human intestine and subjected tcrδ^−/−^ mice to experimental gut injury. In addition, we used polychromatic flow cytometry to calculate percentages of viable IEL (defined as CD3^+^ CD8^+^ CD103^+^ lymphocytes) and the fraction of γδ IEL in surgically resected tissue from infants with NEC and gestational age matched non-NEC surgical controls.

**Results:**

In human preterm infants, the proportion of IEL was reduced by 66% in 11 NEC ileum resections compared to 30 non-NEC controls (p<0.001). While γδ IEL dominated over conventional αβ IEL early in gestation in mice and in humans, γδ IEL were preferential decreased in the ileum of surgical NEC patients compared to non-NEC controls (50% reduction, p<0.05). Loss of IEL in human NEC was associated with downregulation of the Th17 transcription factor retinoic acid-related orphan nuclear hormone receptor C (RORC, p<0.001). TCRδ-deficient mice showed increased severity of experimental gut injury (p<0.05) with higher TNFα expression but downregulation of IL17A.

**Conclusion:**

Complimentary mouse and human data suggest a role of γδ IEL in IL17 production and intestinal barrier production early in life. Specific loss of the γδ IEL fraction may contribute to NEC pathogenesis. Nutritional or pharmacological interventions to support γδ IEL maintenance in the developing small intestine could serve as novel strategies for NEC prevention.

## Introduction

A critical, yet understudied, area in neonatology is the development of intestinal immune regulation in preterm infants, who are prone to exaggerated inflammatory host responses to bacterial antigens [1]. One example is necrotizing enterocolitis (NEC), a common, potentially lethal disease, primarily affecting preterm infants. Epidemiologic studies indicate that NEC incidence peaks at 32 weeks postmenstrual age, suggesting that there is a developmental window of susceptibility [2], [3]. NEC is characterized by uncontrolled intestinal inflammation that can culminate in bowel necrosis [4]–[6]. Approximately 9,000 infants develop NEC in the United States each year, with reported mortality rates of 10–50% [7], [8].

Intraepithelial lymphocytes (IEL) bearing the T cell receptor (TCR) γδ (γδ IEL) are the first type of T cell to colonize the epithelium during embryogenesis providing important immunoprotective and immunoregulatory activities in the perinatal period when conventional TCRαβ T cell responses are not yet fully mature [9]. While the precise role of γδ IEL is not yet clearly defined, they appear to be critical for the maintenance of epithelial integrity through antibacterial defense, tight junction preservation, recognition of epithelial stress, regulation of inflammatory responses and epithelial growth factor production [10]–[15]. The postnatal development of γδ IEL in the human preterm intestine is unknown. Given the immaturity of the intestinal epithelial barrier and its postulated role in NEC [16]–[20], we hypothesized that the developmental regulation of γδ IEL may relate to the window of NEC susceptibility in preterm infants and could represent a new target for disease prevention.

Here we report that γδ IEL are developmentally the prominent IEL subtype in the immature murine and human gut. However, we observed a specific reduction of γδ IEL proportions in the preterm ileum of NEC patients compared to gestational age matched preterm intestine resected for other indications. Loss of γδ IEL resulted in more severe experimental gut injury and inhibited gene expression of IL17 in mice while IEL reduction in human samples correlated with downregulation of IL17 transcription factor RORC. This first report on γδ IEL in the preterm gut suggests a novel target for prevention of severe intestinal complications of prematurity.

## Materials and Methods

### Ethics Statement

Fresh ileum tissue specimens from infants with NEC or non-NEC diagnoses were provided from the Vanderbilt Children's Hospital pathologist under a protocol approved by the Vanderbilt University Institutional Review Board. Informed consent was waived because all samples were de-identified and only demographic data pertinent to the study design (diagnosis and indication for tissue resection, age at time of tissue resection, gestational age, and sex) were collected from patient records prior to tissue release.

C57BL/6J and TCRδ-deficient (tcrδ^−/−^) mice (originally obtained from Jackson laboratories) were bred at an animal facility at Emory University and all studies were approved by the Emory University Institutional Animal Care and Use Committee (IACUC).

### Isolation of human intraepithelial lymphocytes

Patient demographics and surgical indications for the non-NEC control tissues are shown in Table 1 and Table 2 respectively. All samples (NEC and controls) were from the ileum and patients were matched for gestational age. We isolated IEL from surgical ileum specimens as previously described [21]. Briefly, the dissected mucosa was washed in HBSS media without Ca^2+^ and Mg^2+^ containing antibiotics, 5 mM EDTA (Sigma-Aldrich, St. Louis, MO), and 5% heat-inactivated fetal bovine serum (Atlanta Biologicals, Lawrenceville, GA)] for 20 min on a gentle rocker at room temperature. Cell suspensions were pelleted from the supernatant and washed twice in complete HBSS prior to counting using trypan blue exclusion. Cells were resuspended in freezing medium containing 50% Dulbecco's Modified Eagle's medium (DMEM), 40% heat-inactivated fetal bovine serum, and 10% dimethyl sulfoxide (DMSO) (Merck KGaA, Darmstadt, Germany). Cells were frozen in liquid nitrogen for storage until analysis at a concentration of approximately 1×10^6^ cells/ml.

**Table 1:** Demographics of NEC and control patients.

Acute NEC		Controls	
N = 11		N = 30	P values
Gestational age (weeks)	26.9 (25.4; 28.4)	27.0 (26.0; 3.6)	.330
Age (days)	30.0 (17.0; 54.0)	50.0 (5.3; 71.8)	.487
Postmenstrual age (weeks)	32.0 (30.9; 35.5)	37.0 (33.2; 39.6)	.065
Female	36.4% (4)	53.3% (16)	.484

Continuous data are summarized with median (quartiles), and categorical data with percent (frequency). P values are computed with Wilcoxon rank sum test (continuous) and Fisher's exact test (categorical).

**Table 2:** Origins of control small intestinal tissue samples.

	N
Reanastomosis after NEC surgery	9
Resection for congenital intestinal atresia	7
Spontaneous intestinal perforation (SIP)	5
Reanastomosis after SIP repair	4
Reanastomosis after congenital volvulus repair	2
Stricture removal after medical NEC	2
Reanastomosis after gastroschisis repair	1
**Total**	30

### Flow cytometric analysis and sorting of IEL

We performed 7-color flow cytometric analysis of IEL using an LSRII flow cytometer (BD). IEL were thawed and washed in PBS and counted prior to staining with a PE-TexasRed-conjugated amine viability dye (Invitrogen, Grand Island, NY) for 20 min at room temperature in the dark. Cells were then washed with FACS buffer [(PBS containing 1% bovine serum albumin (Sigma-Aldrich) and 0.1% sodium azide (Sigma-Aldrich)] and stained with titrated amounts of PE-TexasRed-conjugated anti-CD14 and anti-CD19 (“dump channel”) (Invitrogen), PerCp-Cy5.5-conjugated anti-CD3 (BD), PE-Cy7-conjugated anti-CD8 (BD), PE-Cy-5 (Tricolor)-conjugated CD103 (Invitrogen), FITC-conjugated anti-TCRαβ (BD), PE-conjugated anti-TCRγδ (BD), and APC-conjugated anti-RORC (eBioscience, clone AFKJS-9). Flow data were analyzed with FlowJo software version 9.3 (Tree Star, Ashland, OR). IEL were identified as CD3^+^, CD103^+^, CD8^+^ cells and characterized as γδ IEL if cells were also TCRγδ^+^ and TCRαβ^−^. To confirm the purity of the IEL populations, we performed flow cytometry analysis on the remaining tissue following IEL preparation (lamina propria cells) and did not detect any CD103^+^ TCRγδ^+^ cells. We only analyzed viable surgical margins with adequate numbers of viable lymphocytes (“dump channel negative”). All flow cytometric gating/analysis was confirmed by an immunologist (MTR) who was blinded to the sample origin. Fluorescent Minus One (FMO) was used to control for nonspecific signal.

### Human RORC and occludin gene expression

Total RNA was extracted from 25 mg of either fresh NEC and non-NEC ileum using the RNeasy Mini Kit or from six 10-micron sections of formalin-fixed, paraffin-embedded tissue pieces using the RNeasy FFPE Kit (Qiagen Valencia, CA). Total RNA was reverse transcribed using the RT^2^ First Strand Kit (Qiagen) per manufacturer's instructions. The cDNA-containing reaction mixture was added to each well of a 96-well-plate PCR array for quantitative real-time (RT) PCR (RORC: Th17 for Autoimmunity and Inflammation PCR Array, occludin: cat no. PPH02571B RT2 Profiler PCR Array; Qiagen). PCR cycles were performed according to the manufacturer's instructions. Expression levels of cytokine genes were quantified using quantitative RT-PCR analysis based on intercalation of SYBR® Green on an ABI 7300 Real-Time PCR system (Life Technologies, Carlsbad, CA). The relative level of mRNA expression for each gene in each sample was normalized to the expression level of reference gene GAPDH and the data were analyzed using the ΔΔC_t_ method [22].

### Human immunohistochemistry

Immunohistochemistry of IEL in formalin-fixed paraffin-embedded tissue sections was performed as recently described [23]. Briefly, 5 µm paraffin embedded sections were cut and placed on charged slides. After epitope retrieval and protein blocking, slides were incubated for 20 minutes with anti-human CD3 (1∶125) (DakoCytomation). A streptavidin-biotin detection system was used followed by application of DAB. The murine Envision+ System, DAB/Peroxidase (DakoCytomation) was employed to produce localized, visible staining. The slides were counterstained with hematoxylin, dehydrated, and cover-slipped.

### Intestinal injury model

To induce intestinal injury, we injected 2 weeks old C57BL/6J or TCRδ-deficient mice with 100 µg/kg platelet activating factor (PAF, Sigma Aldrich, St. Louis, MO) and 1 mg/kg *E. coli* 0128:B12 lipopolysaccharide (LPS, Sigma Aldrich) intraperitoneally as previously reported [24]–[26]. Control animals were injected with PBS vehicle control. Pups were sacrificed two hours later and the distal small intestine was isolated. A portion of the distal small intestine was fixed in 10% formalin (Fisher Scientific, Pittsburgh, PA) for paraffin embedding, sectioning and hematoxylin and eosin (H&E) staining for intestinal injury severity scoring (see below). The remainder was collected in Trizol (Invitrogen, Grand Island, NY) for RNA isolation analysis of cytokine gene expression (see below).

### Murine IEL isolation and analysis

To examine the ontogeny of γδ IEL, small intestines were harvested from 1 week old, 2 weeks old, 3 weeks old, and adult mice (6–8 weeks old). To examine frequencies of γδ IEL in mice subjected to experimental intestinal injury, small intestines were harvested from 2 weeks old mice subjected to experimental intestinal injury as described above. Intestines were cut longitudinally and rinsed of luminal contents and subequently cut into 1 cm pieces and shaken at 250 rpm for 20 min at 37°C in HBSS (Ca/Mg-free) with 5% fetal bovine serum and 2 mM EDTA. The cell suspensions were passed through a 100 µm cell strainer then through glass wool columns and centrifuged at 1500 rpm. The cell pellets were resuspended in 45% isotonic Percoll, underlain with 70% Percoll, and centrifuged at 2000 rpm for 25 min. The IEL at the interface of 44% and 70% Percoll were collected and washed for flow cytometric analysis. This technique for IEL isolation has been shown to be valid for both neonatal and adult murine intestines [27], [28].

Surface staining was performed at 4°C for 20 min in PBS with 5% FBS. Fc receptors were blocked with anti-FcγRIII/II (2.4G2) and the following antibodies were used: APC-conjugated TCRβ (clone H57–597), PE-Cy7-conjugated CD3ε (clone 145-2C11) AlexaFluor 700-conjugated CD4 (clone RM4–5) from eBioscience; PE-Texas Red-conjugated CD8α (clone 5H10) from Invitrogen; FITC-conjugated anti-TCRγδ (clone UC7-13D5), PerCP-Cy5.5-conjugated CD4 (clone RM4–5), and PE-conjugated CD103 (clone M290) from BD Pharmingen.

To examine relative frequencies of γδ IEL in wild-type mice subjected to intestinal injury, IEL were isolated from dam-fed wild-type 2 week-old mice or mice subjected to intestinal injury as described above. IEL were subsequently isolated, stained, and flow cytometric analysis was conducted on a BD LSR II (BD Biosciences, Franklin Lakes, NJ). IEL were defined as CD103^+^, CD3ε^+^ and characterized as γδ IEL if cells were also TCRγδ^+^ and TCRβ^-^.

### Intestinal injury scoring

H&E sections were scored by a blinded reviewer on a 5 point scale: grade 0: no injury; grade 1: mild intestinal dilatation, mild submucosal edema or lamina propria separation, epithelial apoptosis; grade 2: moderate submucosal or lamina propria separation, submucosal edema, epithelial sloughing or necrosis, epithelial mucus depletion; grade 3: severe submucosal or lamina propria separation, severe edema, villous sloughing; grade 4: severe villous sloughing, transmural necrosis. This scale is a compilation of scales used in similar intestinal injury models [20], [25], [29]–[32].

### Murine mRNA isolation and cytokine gene expression

Distal small intestinal samples were homogenized and total RNA isolated and reverse transcribed from random hexamer primers using the QuantiTect Reverse Transcription Kit (Qiagen, Carol Stream, IL). The resulting cDNA products were analyzed by real-time quantitative RT-PCR (iQ SYBR Green Supermix on MyiQ real time PCR detection system, Biorad, Hercules, CA) for IL17A, TNFα and GAPDH mRNA). The relative level of mRNA expression for each gene in each sample was normalized to the expression level of reference gene GAPDH and the data were analyzed using the ΔΔC_t_ method [22].

Primer information:

GAPDH-forward: TGG CAA AGT GGA GAT TGT TGC C

GAPDH-reverse: AAG ATG GTG ATG GGC TTC CCG

IL17A-forward: CAG CAG CGA TCA TCC CTC AAA G

IL17A-reverse: CAG GAC CAG GAT CTC TTG CTG

TNFα-forward: CTA CTC CCA GGT TCT CTT CAA

TNFα-reverse: GCA GAG AGG AGG TTG ACT TTC

### Statistical analysis

#### Human studies (Vanderbilt)

Gene expression and flow cytometry cell type data followed skewed distributions and underwent logarithmic transformation. Data were compared between independent groups using Student's t test. Lamina propria lymphocytes (LPL) and IEL RORC gene expression from the same set of subjects were compared using the paired t test. Associations between TCRγδ IEL and RORC mRNA expression and age parameters were explored using Pearson's correlation coefficient after logarithmic transformation of the skewed variables. The relationship between the proportion of TCRγδ^+^ IEL and gestational age in non-NEC surgical control samples followed a non-linear distribution. Thus, a model was fitted to a second order polynomial equation using non-linear regression and plotted with 95% confidence bands. Goodness of fit was evaluated by the R^2^ parameter. The runs test was performed to determine whether the curve deviated systematically from the data.

#### Animal studies (Emory)

Data are reported as mean ± standard error of the mean (SEM). Statistical differences were determined by one-way analysis of variance (ANOVA) or Student's t test as appropriate. A p<0.05 was considered significant.

## Results

### Surgical ileal mucosa from NEC patients was marked by decreased proportions of IEL and TCRγδ IEL ratios compared to non-NEC surgical controls

To determine whether γδ IEL may play a protective role against intestinal injury in the premature human intestine, we studied the development, phenotype and distribution of these cells in relationship to total viable CD3^+^ CD8^+^ T cells in surgical ileum samples. We prospectively isolated IEL from fresh tissue obtained through medically indicated surgical resection for 11 NEC and 30 non-NEC patients. All tissue sections were ileum and were from infants of comparable gestational age (GA) (p = 0.330), age (p = 0.487), postmenstrual age (PMA) (p = 0.065), and sex distribution (p = 0.484) (Table 1). Non-NEC cases included resections for reanastomoses for various surgical indications (16), congenital intestinal bowel obstruction (7), spontaneous (focal) intestinal perforation (5), and tissue from stricture removal after medical NEC (2) (Table 2). Median mucosa weights for NEC and non-NEC tissues were similar (310 mg and 370 mg, respectively, p = 0.478).

**Figure 1:**
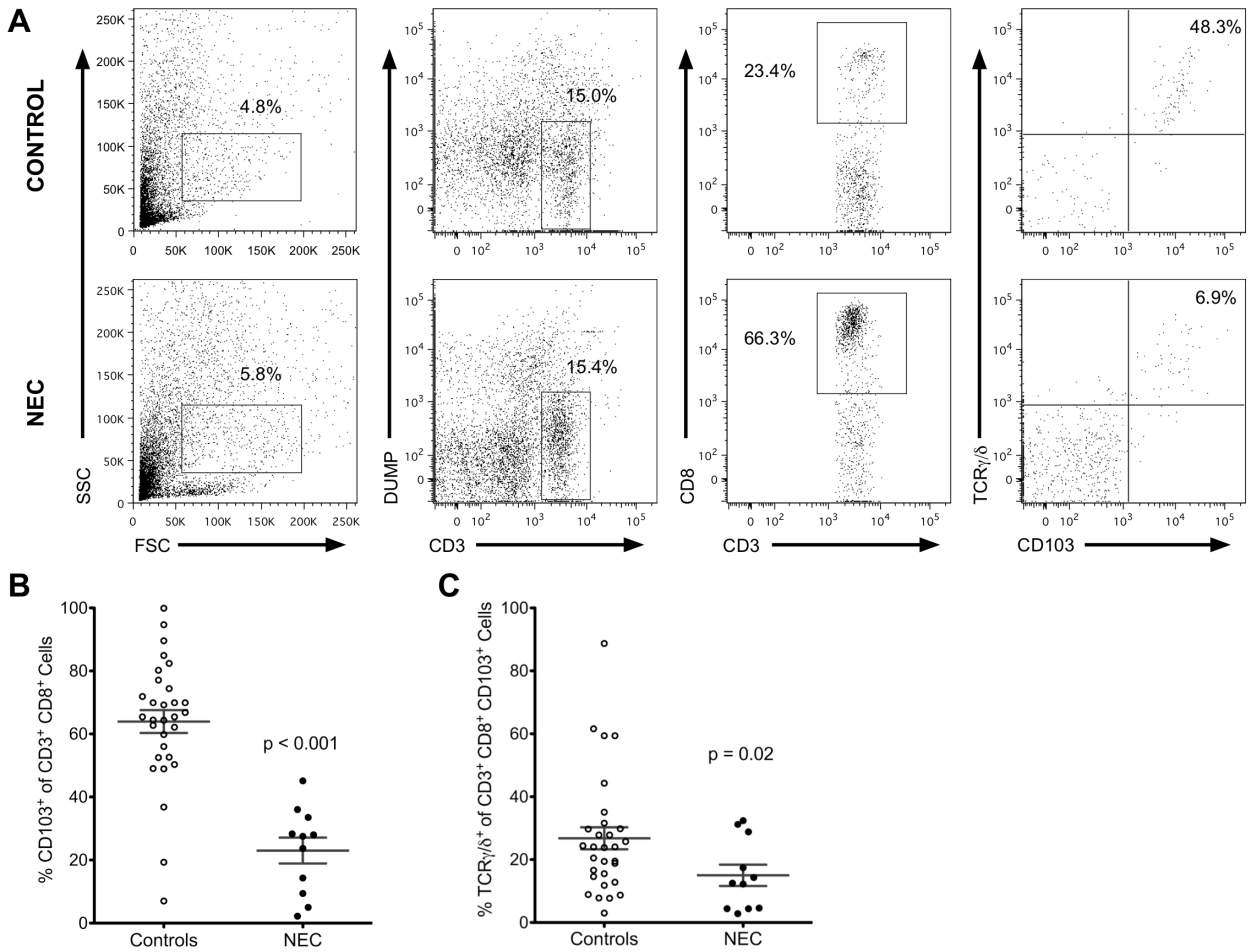
Reduced proportions of γδ IEL subsets in patients with NEC compared to non-NEC surgical controls. (A) Example of the gating strategy used to calculate proportions of γδ IEL subsets. The control sample shown is from a 4 days old 26 weeks gestation infant with spontaneous (focal) intestinal perforation and the NEC sample is from a 15 days old 28 weeks gestation infant with surgical NEC. Gates were set on “live”, CD14^−^, CD19^−^ (“Dump” negative) and CD3^+^ cells before applying to sub-populations. Next we identified CD3^+^ CD8^+^ T cells followed by differentiating conventional CD3^+^ CD103^+^ TCRαβ from TCRγδ IEL (γδ IEL). The patient with NEC showed significant reduction in γδ IEL with a corresponding greater proportion of αE integrin (CD103) negative, conventional T cells. Dot plot of total IEL (B) and γδ IEL (C) proportions were statistically significantly reduced in NEC tissue compared to non-NEC controls, p<0.001 and p = 0.02, respectively.

We compared the proportions of total IEL and γδ IEL as demonstrated in Figure 1A. Using flow cytometry we defined IEL as life CD3^+^ CD8^+^ CD103^+^ lymphocytes and characterized as γδ IEL if cells were also TCRαβ^-^ and TCRγδ^+^. Compared to non-NEC surgical controls, NEC samples exhibited significantly lower numbers of total IEL (mean 2,342 versus 124 cells per tissue section, p<0.01). Because NEC is associated with necrosis and intestinal epithelium loss likely explaining reduction in total IEL, we calculated percentages of IEL based on total CD3^+^ CD8^+^ cells isolated in tissue epithelium preparations. The mean fraction of IEL within epithelial CD3^+^ CD8^+^ cells in non-NEC surgical controls was 64% compared to 23% in NEC, Figure 1B, p<0.001). Within the IEL compartment of the control group, a sizable proportion of cells were γδ IEL (mean 27%), which was significantly decreased in NEC patients (mean 15%) (Figure 1C, p = 0.02). Therefore surgical NEC was characterized by a preferential reduction in γδ IEL over αβ IEL.

We considered the possibility of sample contamination from conventional lymphocytes in the lamina propria. We performed flow cytometry analysis on the remaining lamina propria tissue (LPL) following IEL preparation and did not detect any CD103^+^ TCRγδ^+^ cells supporting the purity of IEL and LPL preps. In addition, the mean total number of viable CD3^+^ cells isolated from the epithelium of NEC samples was 50% of cells identified in non-NEC samples (5,128 vs. 10,228 cells, p = 0.189), suggesting that the reduced IEL fraction in NEC is not explainable by significant influx of CD3^+^ cells from other compartments.

### γδ IEL are the predominant IEL subtype in the immature murine and human small intestine

**Figure 2:**
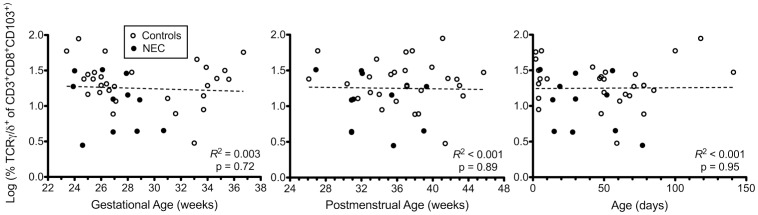
Developmental regulation of γδ IEL subsets in humans. Logarithmic transformed percentages of γδ IEL were plotted against gestational age (GA), postmenstrual age (PMA  =  gestational age plus chronological age) and age. Using Pearson's correlation coefficient we did not detect any association of γδ IEL proportions with GA, PMA or age in either NEC or non-NEC control patients.

**Figure 3:**
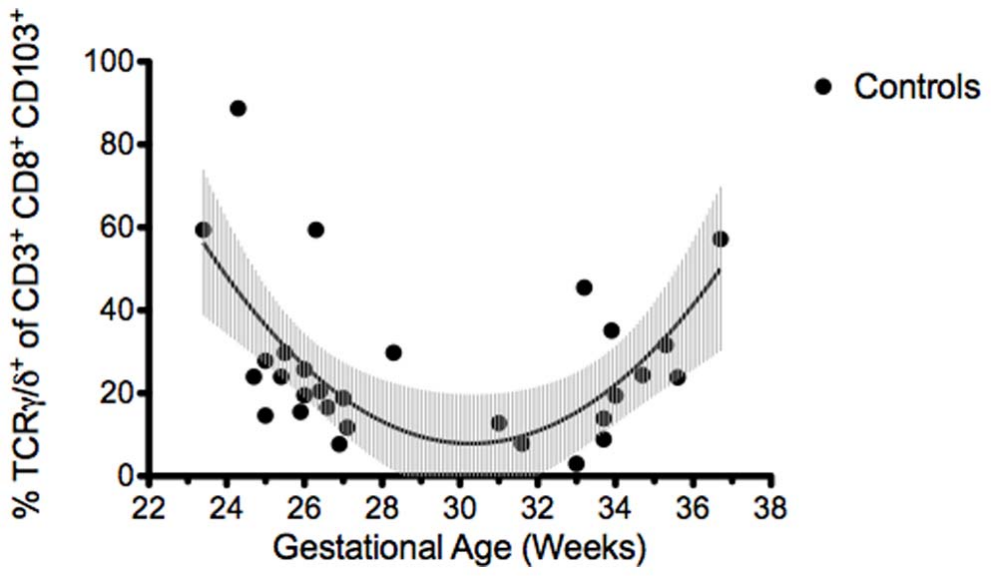
Window of susceptibility with low γδ IEL subsets in human neonates. Graph depicting the relationship between percentage of γδ IEL and gestational age in non-NEC surgical control samples. The black line shows the curve estimate of the U-shaped association as modeled by nonlinear regression with 95% confidence intervals of the model curve shaded in grey. The lowest percentage of γδ IEL occurs between 27 and 32 weeks gestation.

Since NEC predominantly affects preterm infants, we examined whether γδ IEL are developmentally regulated in the preterm intestine. We examined the relationship between γδ IEL proportions and gestational age, postmenstrual age, and age. We did not observe a clear association between γδ IEL proportions and postmenstrual age or postnatal age, suggesting that even the most premature infants contain significant fractions of natural γδ IELs at birth [33] (Figure 2). Interestingly, the relationship between γδ IEL proportions and gestational age in non-NEC surgical control samples followed a U-shaped distribution as determined by nonlinear regression. This model accounted for 37% of the variance of the data (R^2^ = 0.37). The observed data did not deviate significantly from the model curve as determined by the runs test (p = 0.31). This distribution suggests a possible window of vulnerability for NEC across gestation (Figure 3).

**Figure 4:**
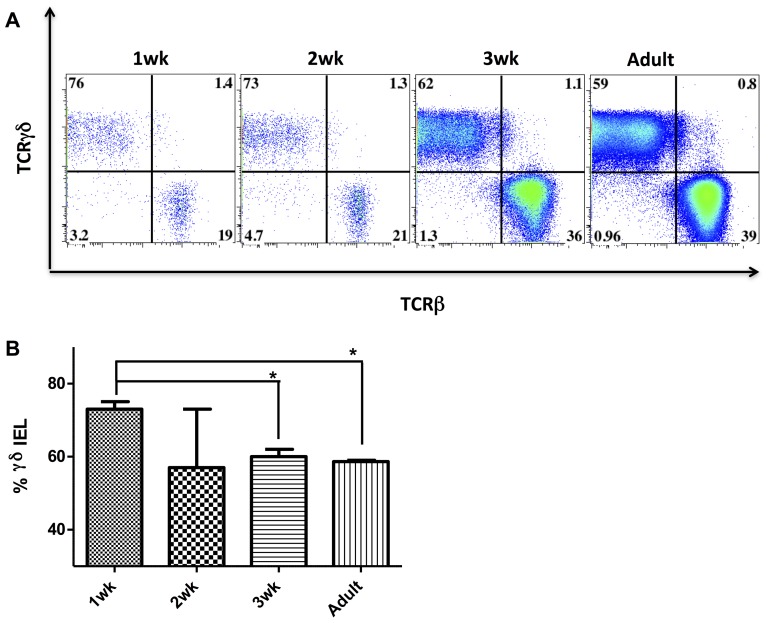
γδ IEL are the predominant IEL subtype in the immature murine small intestine. A) Flow cytometry of distal small intestinal intraepithelial cells from 1 week old (1 wk, n = 4), 2 weeks old (2 wk, n = 3), 3 weeks old (3 wk, n = 2) and adult (n = 1) C57BL/6J mice stained for CD3, CD8α, CD103, TCRγδ and TCRβ as described. Intraepithelial cells were pregated on CD103^+^, CD3^+^ to depict IEL and then further gated on TCRγδ and TCRβ as shown. B) Percent (mean ±SE) γδ IEL (defined as percent of total IEL that were TCRγδ^+^, TCRβ^−^ IEL) in the distal small intestines of 1 wk, 2 wk, 3 wk, and adult mice. Data are representative of 3 independent experiments (*p<0.05 when compared to 1 wk samples).

Young mice are frequently used for NEC-like injury models and correlating the maturity of the mucosal immune system between neonatal mice and humans is complex [33]. In addition, the human data on postnatal development may have been skewed, as neonatal intestinal tissue samples cannot be obtained from healthy neonates. Therefore we isolated epithelial-associated immune cells from the small intestines of wild type neonatal mice ages 1 week to adult (Figure 4A). γδ IEL were the predominant IEL subtype in younger mice (73% in 1 week old mice versus 59% in adult mice, p<0.05), with frequency approaching adult levels by 3 weeks of life (60%, p<0.05 vs. 1 week old) (Figure 4B).

### Intestinal injury in wild-type mice is not associated with a selective reduction in γδIEL

**Figure 5:**
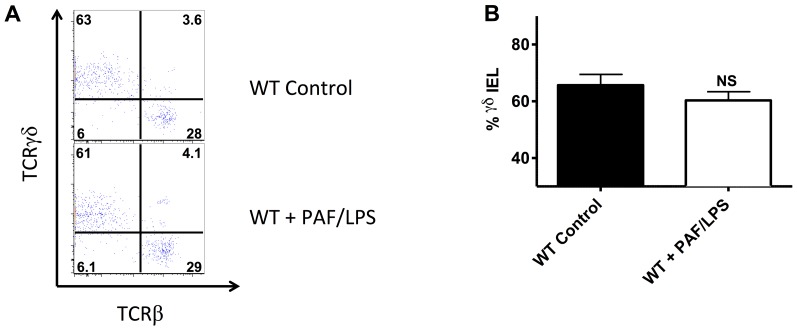
Intestinal injury in wild-type mice is not associated with a selective reduction in γδ IEL. A) Flow cytometry of small intestinal intraepithelial cells isolated from 2 weeks old dam fed wild-type (WT control, n = 2) or wild-type mice subjected to experimental gut injury as described (WT^+^PAF/LPS, n = 2). C57BL/6J mice stained for CD103, CD3, CD8α, TCRγδ and TCRβ as described above. Intraepithelial cells were pregated on CD103^+^, CD3^+^ to depict IEL and then further gated on TCRγδ and TCRβ as shown. B) Percent (mean ±SE) γδ IEL (defined as percent of total IEL that were TCRγδ^+^, TCRβ^-^ IEL). Data are representative of 3 independent experiments (NS indicates no statistical difference between groups).

For ethical reasons, it is not possible to determine definitively whether the selective reduction of γδ IEL in human NEC occurred prior to or as a result of intestinal injury. Therefore we sought to determine whether experimental intestinal injury in a murine model causes selective reduction in γδ IEL. To induce intestinal injury, we injected 2 weeks old C57BL/6J or TCRδ^−/−^ mice intraperitoneally with 100 µg/kg PAF and 1 mg/kg *E. coli* 0128:B12 LPS or PBS vehicle control as described above. Pups were sacrificed two hours later and small intestinal epithelial-associated immune cells were isolated as stated above. We detected no differences in percentages of γδ IEL between control mice and those subjected to experimental intestinal injury (Figure 5). These data suggest that the selective reduction in γδ IEL associated with human NEC is not a secondary finding following injury but may indicate a specific risk factor.

### Significant reduction in RORC expression in NEC tissue correlates with reduction of IEL

**Figure 6:**
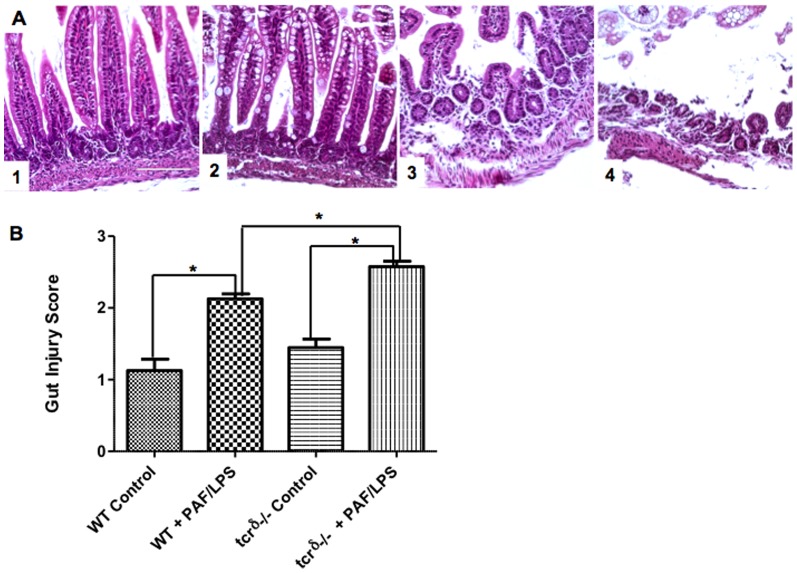
Retinoic acid-related orphan nuclear hormone receptor C (RORC) gene expression in intestinal lymphocyte subsets. (A) We measured gene expression levels of RORC by quantitative RT-PCR in 15 NEC tissue sections and 7 controls by quantitative RT-PCR array. RORC gene expression was significantly decreased in NEC samples versus controls (p<0.001). Relative level of mRNA expression of RORC in each sample was normalized to the expression level of reference gene GAPDH. (B) To determine whether loss of IEL contributed to reduction of RORC expression, we compared IEL with lamina propria lymphocytes (LPL) from the same tissue source using 10 non-NEC controls. RORC gene expression was significantly higher in IEL compared to LPL (p = 0.01). (C) RORC gene expression and proportions of total TCRγδ IEL correlated positively with each other (p = 0.02).

TCRγδ cells have been attributed an important role in innate mucosal immune responses, partially mediated through the production of IL17 [35], [36]. TCRγδ IEL have been specifically shown to produce IL17 under inflammatory conditions [37], [38]. To determine whether a similar mechanism may play a role in the human neonatal gut, we measured the gene expression of retinoic acid-related orphan nuclear hormone receptor C (RORC) in the small intestinal mucosa of 15 NEC patients compared to 7 surgical controls. Human RORC is an analogue to the murine retinoid orphan receptor (RORγt), which drives expression of IL17 in γδ IEL [36]. Since expression of IL17 is dependent on cell stimulation and IEL numbers were too low to isolate sufficient cells for stimulation assays, we used RORC gene expression as a correlate for IL17 production [39]. By quantitative RT-PCR, RORC gene expression in NEC samples was reduced by a median of 10 fold (p<0.001, Figure 6A). Next, we sought to determine if the reduction of RORC expression in NEC could be explained by loss of γδ IEL. We measured RORC gene expression in LPL and IEL isolated from identical tissue sections from non-NEC controls. RORC gene expression was significantly higher in IEL compared to LPL (p = 0.01, Figure 6B). In addition, we found a statistically significant positive correlation between total TCRγδ^+^ IEL proportions and RORC gene expression (Pearson R^2^ = 0.41, p = 0.02 (Figure 6C). Cumulatively, these data suggest that loss of γδ IEL in NEC may limit intestinal barrier defense through decreased production of IL17.

### Intestinal injury in TCRδ-deficient mice is associated with increased TNFα but decreased IL17A gene expression

**Figure 7:**
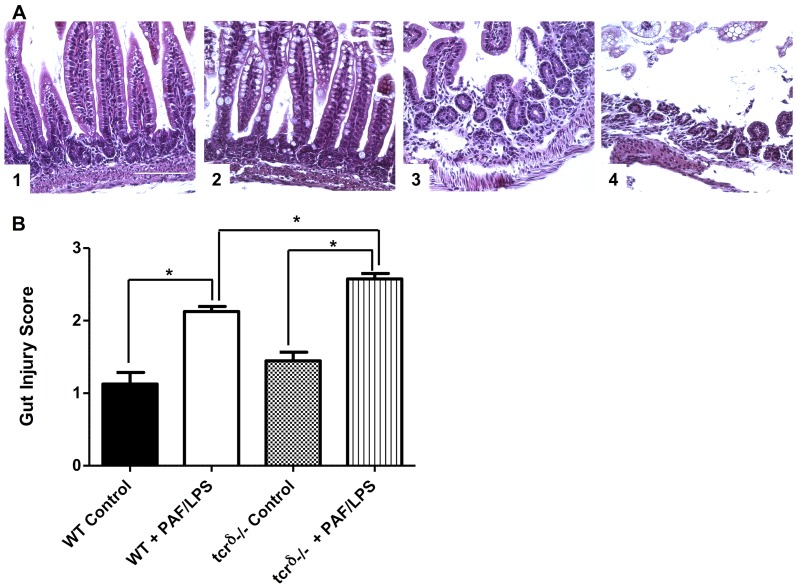
γδ T cells reduce experimental gut injury. A) Representative H&E staining of distal small intestines isolated from dam fed wild-type (1) or TCRδ^−/−^ (3) mice with normal histologic appearance; or wild-type (2) or TCRδ^−/−^ (4) mice subjected to experimental gut injury (PAF/LPS) as described (scale marker  = 100 µm). Note shortened villi and epithelial sloughing with inflammatory infiltrate in wild-type PAF/LPS mice (2) and submucosal edema with severe villous sloughing in TCRδ^−/−^ PAF/LPS mice (4). B) Histologic severity score (mean ±SE) of distal small intestinal sections obtained from dam fed wild-type (WT control) or TCRδ^−/−^ (tcrδ^−/−^ control) mice; or wild-type (WT PAF/LPS) or TCRδ^−/−^ (tcrδ^−/−^ PAF/LPS) mice subjected to experimental gut injury as described. Data are representative of 4 independent experiments with at least 3 mice per condition per experiment (*p<0.05).

**Figure 8:**
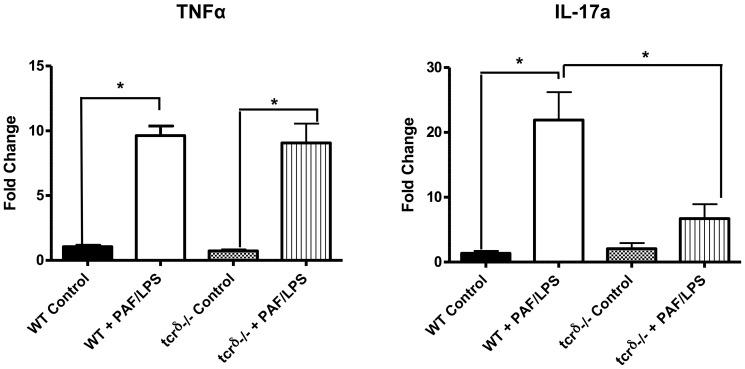
More severe intestinal injury in TCRδ^−/−^ mice was associated with increased TNFα and decreased IL17A gene expression. Gene expression of TNFα and IL17A as measured by quantitative RT-PCR in distal small intestinal sections obtained from dam fed wild type (WT control) or TCRδ^−/−^ (tcrδ^−/−^ control) mice; or wild type (WT PAF/LPS) or TCRδ^−/−^ (tcrδ^−/−^ PAF/LPS) mice subjected to experimental gut injury injury as described. Data are representative of 3 independent experiments with at least 3 mice per condition per experiment (*p<0.05).

To investigate the role of γδ IEL in mucosal homeostasis and cytokine response, we measured mRNA expression of intestinal TNFα and IL17A in mice lacking γδ IEL and exposed to experimental gut injury as described above. At baseline, there was no difference in the histologic appearance of control dam fed wild type or TCRδ^−/−^ mice (Figure 7A). When subjected to experimental gut injury, *TCR*δ^−/−^ mice were found to have significantly worse disease scores compared to wild type mice (2.1±0.1 versus 2.5±0.1, p<0.05) (Figure 7B). TCRδ^−/−^ mice also exhibited increased incidence of injury (defined as severity scores >2) when compared to wild-type mice (59% vs. 29%). Similarly, intestinal TNFα and IL17A mRNA expression was low in the steady state. In response to PAF-induced epithelial injury, intestinal mRNA expression of both TNFα and IL17A increased in wild type mice. Interestingly, TCRδ-deficient mice demonstrated significantly reduced expression of IL17A (7-fold versus 22-fold induction in IL17A expression, p<0.05) (Figure 8). These data suggest that epithelial injury may induce TCR γδ T cells to express IL-17 in order to protect the intestinal barrier.

### Occludin gene expression is decreased in NEC tissue

**Figure 9:**
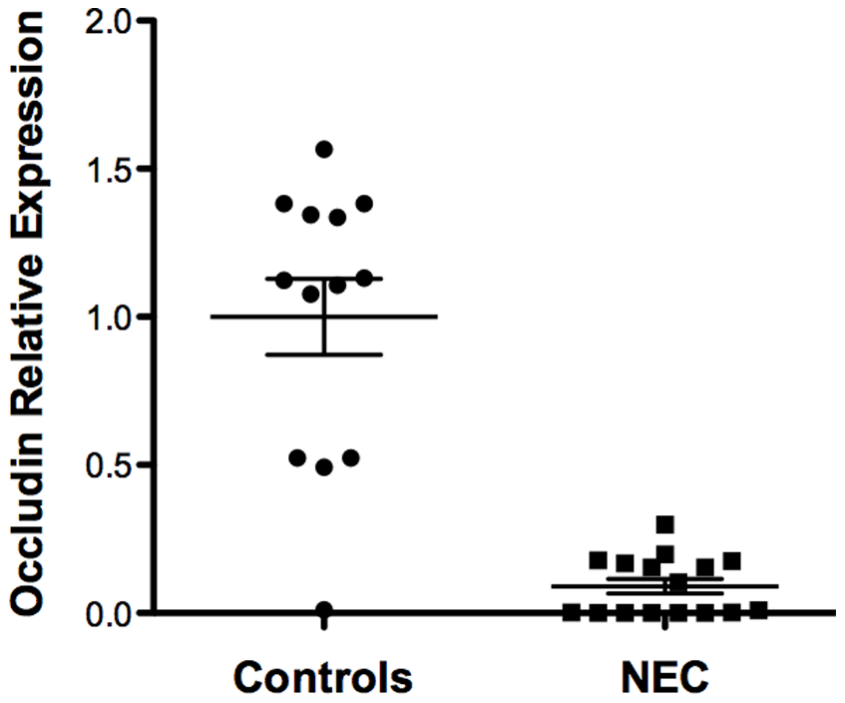
Occludin gene expression in human NEC versus control mucosa. (A) We measured gene expression levels of occluding by quantitative RT-PCR in 16 NEC tissue sections and 13 controls by quantitative RT-PCR array. Occludin gene expression was significantly decreased in NEC samples versus controls (p<0.0001). Relative level of mRNA expression of occludin in each sample was normalized to the expression level of reference gene GAPDH.

Occludin forms rings at sites of γδ IEL/epithelial contact and promotes γδ IEL migration into epithelial monolayers [40]. Enterocytes internalized occludin in experimental NEC but expression in human NEC was unchanged in the small intestine by immunohistochemistry [16]. We sought to determine occludin expression in human NEC tissue to test the possibility that reduced expression may inhibit migration of γδ IEL into the intraepithelial compartment. We found statistically significant reduction in occludin gene expression in by quantitative RT-PCR in 16 NEC tissue sections compared to 13 controls (p<0.0001, Figure 9).

## Discussion

Although the exact biological function of γδ IEL is elusive, these cells reportedly play an important role in innate mucosal immune responses by preventing invasion of pathogenic bacteria [41], partially mediated through the production of IL17 [35], [36]. In addition, γδ IEL maintain epithelial barrier function through production of keratinocyte growth factor in mice [15], [42] and protect from dextran sodium sulfate (DSS) induced colitis [11], [43]. Furthermore, γδ IEL appear to be critical for immune homeostasis [44], [45]. Since epithelial barrier disruption, invasion of pathogenic bacteria and exaggerated inflammation are key contributors to the development of NEC in the preterm infant [46], we sought to determine the developmental regulation of γδ IEL in the small intestinal mucosa of preterm infants and a possible role in NEC pathogenesis. We demonstrate here for the first time abundance of γδ IEL in the preterm gut but also a statistically significant reduction in acute NEC. Different subtypes of γδ IEL exist [34]; however we focused on CD8^+^ γδ IEL, because of their dominance in the small intestine [47]. The loss of CD8^+^ γδ IEL in NEC could represent a disproportional lack of immune regulatory IEL, which may be critical in the phase of precipitously increasing antigen exposure [10].

We do not know the reason for reduced IEL proportions in NEC. We considered the possibility that the reduction of IEL may be due to loss of epithelium through tissue necrosis. However, as shown in Figure S1, analyzed NEC tissue contained epithelium and IEL, although in lower numbers compared to non-NEC controls. We controlled for NEC-associated epithelium loss by calculating the fraction of IEL within the total number of epithelial CD3^+^ CD8^+^ cells. In addition, the preferential reduction of γδ IEL compared to αβ IEL cannot be explained by absence of enterocytes.

We contemplated the possibility of contamination from conventional lymphocytes in the lamina propria. We think this is unlikely since our protocol effectively separates IEL and LPL cells as previously published and shown in Figure 5B [21]. To further confirm the purity of the IEL populations, we performed flow cytometry analysis on the remaining tissue (LPL) following IEL preparation and did not detect any CD103^+^ TCRγδ^+^ cells. We have previously described an increase in non-regulatory T cells in NEC lamina propria [21] and therefore it is possible that reduction in IEL proportions in NEC is due to additional T cells entering the epithelium. However, as described above, non-NEC samples contained twice as many epithelial T cells in as NEC samples making data skew by contaminating cells unlikely. In addition, influx of CD3^+^ cells in NEC would not explain the specific reduction in the γδ IEL fraction.

We wondered if the immature mucosal immune system contributed to the reduced γδ IEL proportions in the small intestine of patients with NEC. While an inverse relationship between number of villus IEL and increasing age has been reported in adults [48], the postnatal developmental regulation of γδ IEL in preterm infants was unknown. We found robust proportions of γδ IEL early in life even at extreme prematurity. In addition, we defined the postnatal development of γδ IEL in human non-NEC infants showing a U-shaped distribution in the last trimester (Figure 3). TCRγδ IEL may be initially recruited to the immature gut as the predominant IEL subtype in order to protect against potential injury at a time when the gut barrier is immature and exposure to new bacterial antigens is rapidly growing [49].

One potential mechanism for the reduced γδ IEL fraction in preterm infants at risk for NEC may be *in-utero* exposure to inflammation. Histological chorioamnionitis with fetal involvement has been considered a possible risk factor for NEC [50] and inflammation associated with this pregnancy complication may lead to occludin endocytosis and therefore reduced migration of γδ IEL into the intraepithelial compartment [39]. Occludin internalization has been reported in experimental NEC [16] and we show that small intestinal occludin gene expression was significantly decreased in NEC tissue compared to non-NEC controls. We consider chorioamnionitis a more likely candidate for γδ IEL reduction than inflammation associated with NEC because our control group included infants with conditions that involved intestinal perforation with a significant inflammatory response.

Homing and/or retention of lymphocytes in the intestinal epithelium is maintained by expression of integrin αEβ7, which is regulated by TGFβ signaling [51], [52]. We recently discovered overexpression of its negative regulator Smad7 in NEC tissue [53]. Inhibited TGFβ signaling reduces expression of integrin αE (CD103), which in conjunction with integrin beta 7 forms a complete heterodimeric integrin molecule that is thought to mediate retention of IEL in the epithelium [54]. Downregulation of TGFβ may also play a role in reduced expression of RORC [55] and enhanced T cell mediated inflammation in NEC tissue [21], [56].

NEC occurs only in a subgroup of preterm infants and its risk is increased with lack of breast milk feeding and a microbiome with decreased diversity [6], [46], [57], [58]. Expansion of intestinal γδ IEL in mice depends on bacterial interaction [36] and the altered microbiome in NEC may contribute to underdevelopment of γδ IEL. Dietary natural aryl hydrocarbon receptor (AhR) ligands are critical for normal intestinal immune development [59] and postnatal maintenance of IEL [60]. Lack of AhR signaling has been implicated in the pathogenesis of inflammatory bowel disease [61]. The role of AhR ligands in maintaining γδ IEL in preterm infants is unknown and should be explored in future studies.

In conclusion, we demonstrate for the first time the postnatal development of γδ IEL in the premature intestine and therefore contribute to the understudied area of human neonatal mucosal immune development [62]. We further show that the normally enriched fraction of γδ IEL in the ileum of premature infants is significantly reduced in surgical NEC. Complimentary animal and human data suggest a potentially important role of γδ IEL in IL17 production and intestinal barrier protection. Ways to recruit and maintain this likely important T cell population in the preterm gut could serve as a novel strategy to reduce or prevent NEC and other intestinal complications originating early in life.

## Supporting Information

Figure S1 **Immunohistochemistry of intraepithelial lymphocytes.** Immunohistochemistry for CD3^+^ cells in representative tissue sections. (A) Eleven days old 32 weeks gestation infant with NEC. (B) Four days old 33 weeks gestation infant with intestinal atresia. Arrows illustrate intraepithelial lymphocytes, which were reduced in NEC patients (200× magnification).
(TIFF)
