# Correction: Small Intestinal Intraepithelial TCRγδ^+^ T Lymphocytes Are Present in the Premature Intestine but Selectively Reduced in Surgical Necrotizing Enterocolitis

**DOI:** 10.1371/journal.pone.0105487

**Published:** 2014-08-06

**Authors:** The PLOS ONE Staff

Figure 6 is incorrect. The authors have provided a corrected version here.

**Figure 6:**
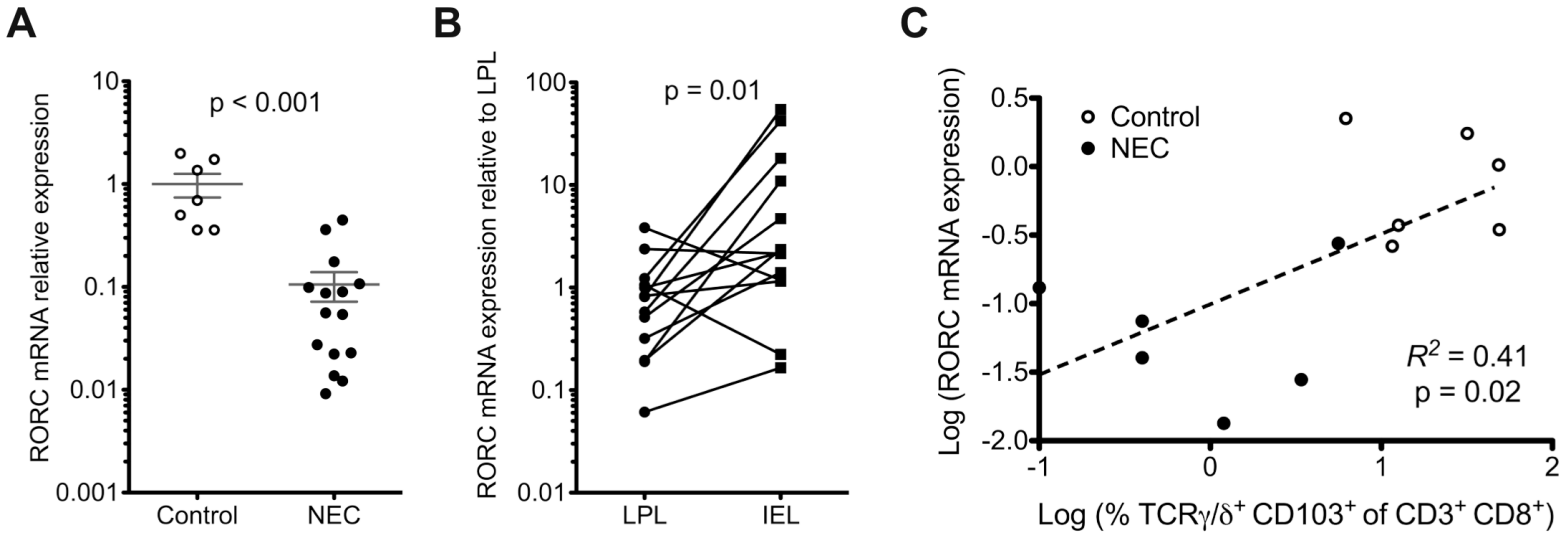
Retinoic acid-related orphan nuclear hormone receptor C (RORC) gene expression in intestinal lymphocyte subsets. (A) We measured gene expression levels of RORC by quantitative RT-PCR in 15 NEC tissue sections and 7 controls by quantitative RT-PCR array. RORC gene expression was significantly decreased in NEC samples versus controls (p<0.001). Relative level of mRNA expression of RORC in each sample was normalized to the expression level of reference gene GAPDH. (B) To determine whether loss of IEL contributed to reduction of RORC expression, we compared IEL with lamina propria lymphocytes (LPL) from the same tissue source using 10 non-NEC controls. RORC gene expression was significantly higher in IEL compared to LPL (p  =  0.01). (C) RORC gene expression and proportions of total TCRγδ IEL correlated positively with each other (p  =  0.02).
