# Supplementary figures and images for: Small Intestinal Intraepithelial TCRγδ+ T Lymphocytes Are Present in the Premature Intestine but Selectively Reduced in Surgical Necrotizing Enterocolitis
Source: PLoS One. 2014 Jun 6;9(6):e99042. doi: 10.1371/journal.pone.0099042 (PMC4048281; doi:10.1371/journal.pone.0099042)

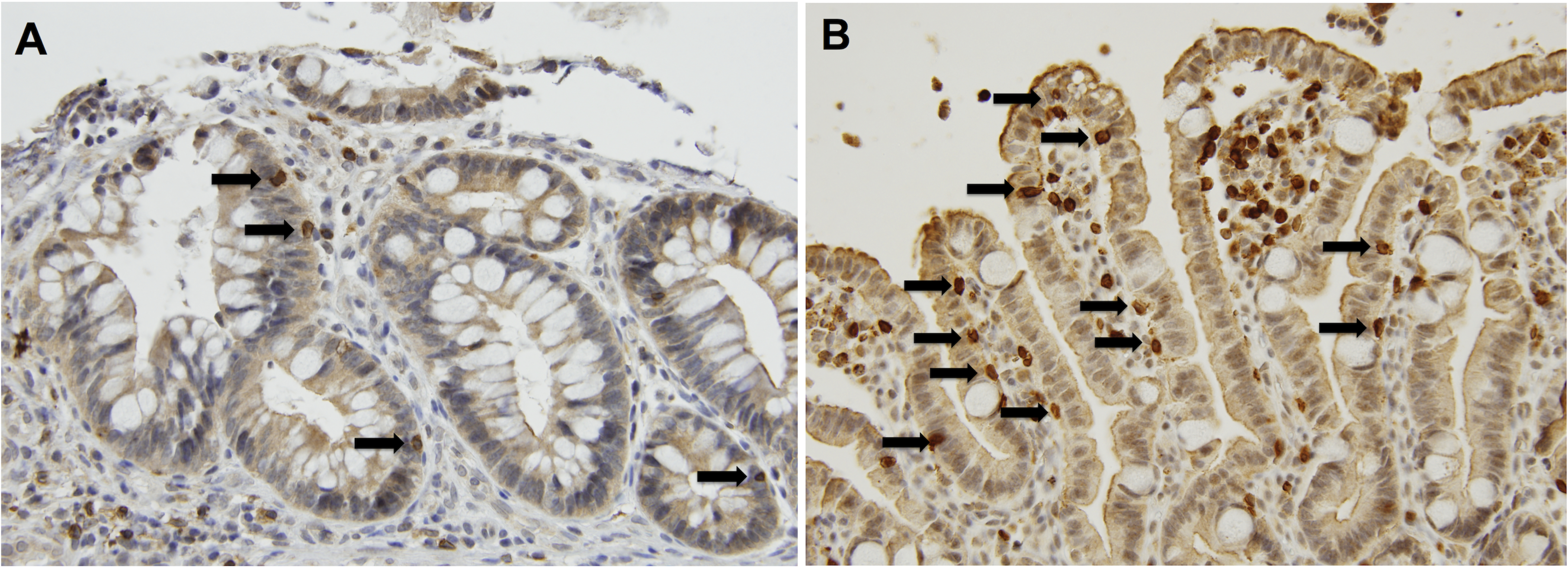

Supplement: Figure S1 — Immunohistochemistry of intraepithelial lymphocytes. Immunohistochemistry for CD3+ cells in representative tissue sections. (A) Eleven days old 32 weeks gestation infant with NEC. (B) Four days old 33 weeks gestation infant with intestinal atresia. Arrows illustrate intraepithelial lymphocytes, which were reduced in NEC patients (200× magnification). (TIFF) [file pone.0099042.s001.tiff]
